# Acyclovir Therapy Reduces the CD4+ T Cell Response against the Immunodominant pp65 Protein from Cytomegalovirus in Immune Competent Individuals

**DOI:** 10.1371/journal.pone.0125287

**Published:** 2015-04-29

**Authors:** Annette Pachnio, Jusnara Begum, Ashini Fox, Paul Moss

**Affiliations:** 1 School of Cancer Sciences, College of Medical and Dental Sciences, University of Birmingham, Birmingham, United Kingdom; 2 Department of Genito Urinary Medicine, Nottingham University Hospitals Trust, Nottingham, United Kingdom; University of Regensburg, GERMANY

## Abstract

Cytomegalovirus (CMV) infects the majority of the global population and leads to the development of a strong virus-specific immune response. The CMV-specific CD4+ and CD8+ T cell immune response can comprise between 10 and 50% of the T cell pool within peripheral blood and there is concern that this may impair immunity to other pathogens. Elderly individuals with the highest magnitude of CMV-specific immune response have been demonstrated to be at increased risk of mortality and there is increasing interest in interventions that may serve to moderate this. Acyclovir is an anti-viral drug with activity against a range of herpes viruses and is used as long term treatment to suppress reactivation of herpes simplex virus. We studied the immune response to CMV in patients who were taking acyclovir to assess if therapy could be used to suppress the CMV-specific immune response. The T cell reactivity against the immunodominant late viral protein pp65 was reduced by 53% in people who were taking acyclovir. This effect was seen within one year of therapy and was observed primarily within the CD4+ response. Acyclovir treatment only modestly influenced the immune response to the IE-1 target protein. These data show that low dose acyclovir treatment has the potential to modulate components of the T cell response to CMV antigen proteins and indicate that anti-viral drugs should be further investigated as a means to reduce the magnitude of CMV-specific immune response and potentially improve overall immune function.

## Introduction

Cytomegalovirus (CMV) is an ubiquitous β-herpesvirus with a seroprevalence of 60–100% within the adult population in different parts of the world [1]. The virus infects a range of cells and establishes a state of latency with intermittent episodes of lytic replication [2–4]. CMV remains a significant cause of morbidity and mortality in immune suppressed individuals, in particular recipients of solid organ or stem cell transplants, but also HIV positive individuals [5–7], whereas infection in healthy individuals is usually asymptomatic due to immune control of viral replication [8]. However a number of studies have documented the extreme immunodominance of CMV which leads to the accumulation of large numbers of CMV-specific CD4+ and CD8+ T cells [9, 10]. Interestingly, the number of these cells increases with age in a phenomenon termed ‘memory inflation’ and there is increasing concern that this expansion may compromise the ability of the immune system to respond to other infections [11, 12]. CMV seropositivity, together with an inversion of the CD4:CD8 ratio, has been identified as an “Immune Risk Phenotype” (IRP) and is associated with increased risk of mortality [13–15]. In addition, CMV has been implicated with vascular dysfunction and recent reports have demonstrated a correlation between high levels of CMV-specific antibody titre and reduced overall survival in different cohorts [16–19]

As such there is interest in the development of approaches that may be used to reduce the magnitude of the CMV-specific immune response in order to improve immune function. One such method might be to use anti-viral drugs to reduce antigen load, with the expectation that the CMV-specific immune response would therefore decline in a manner similar to that seen in patients with HIV infection who are given HAART therapy [20]. However, there has been debate as to the role of antigen in maintenance of the immune response to chronic viral infections with some studies that show that virus-specific immunity is maintained despite a reduction in antigen load [21, 22]. On the other hand, a recent study using valacyclovir in the murine model of CMV infection demonstrated that high dose antiviral treatment does have the potential to prevent memory inflation of virus-specific T cells in ageing mice *in vivo* [23].

In this study we set out to analyse whether it is possible to modify the human T cell immune response against Cytomegalovirus through the use of long term therapy with low dose acyclovir (ACV). ACV is a guanosine-analogue with activity against a range of herpesviruses. It is particularly active against Herpes simplex virus (HSV)-1 and HSV-2, but also has some activity against CMV, although at 30–100 fold reduced efficacy *in vitro* [24, 25]. Indeed the pro-drug valacyclovir is licensed for its role in suppression of CMV reactivation following renal transplantation. Our data reveal that low dose acyclovir does indeed decrease the cytokine response of CD4+ T cells to the late viral protein pp65 and indicates a potential role of this drug as an agent to modulate CMV-specific immunity.

## Materials and Methods

### Subjects

51 patients were recruited from Birmingham Heartlands Hospital of which 24 were CMV-seropositive (median age 41 years; range 21–74 yrs). Patients were receiving long term treatment with acyclovir at a dose of 400mg twice a day for suppression of recurrent herpesvirus (HSV-1 and HSV-2) infection. The treatment duration ranged between 1 and 108 months with a median of 28 months. They had no underlying disease conditions otherwise. 26 age and gender matched healthy CMV-seropositive control subjects were recruited from the same hospital (median age 34 years; range 20–61 yrs). CMV-serostatus was determined by detection of virus-specific IgG antibodies in serum using ELISA (BioCheck Inc. Foster City, CA, USA) according to manufacturer’s instructions. The study was approved by the National Research Ethics Service (West Midlands—Black Country REC ref. 06/Q2702/5) and all individuals gave written informed consent before participation.

### Cell separation, antigen stimulation and intracellular cytokine staining

Peripheral blood mononuclear cells (PBMC) were isolated from heparinised blood samples by density gradient centrifugation using Lymphoprep solution (Axis-Shield, Huntindon, U.K.). The frequency of CMV-reactive T cells was determined by antigen stimulation and subsequent detection of cytokine production. Briefly, fresh PBMC were stimulated with CMV-lysate (strain AD169) or CMV-derived peptides for 16 hours in the presence of BrefeldinA (10μg/ml final concentration; Sigma-Aldrich, Gillingham, UK). Peptide pools contained either pp65 or IE-1 derived peptides (all Alta Biosciences, Birmingham, UK) and were used at a final concentration of 1μg/ml per peptide. As a positive control cells were stimulated with staphylococcus enterotoxin B (SEB) (0.2 μg/ml final concentration; Sigma-Aldrich) and unstimulated cells served as a negative control. As an additional control 8 CMV seronegative donors were recruited and PBMC were stimulated with peptide pools in the same way. No cytokine responses were observed from this population. For PPD a final concentration of 10μg/ml was used to stimulate PBMC and Adenovirus 5 lysate was used at a concentration of 60μg/1x10^6^ cells. Cells were then stained with αCD4-PE (BD Biosciences, Oxford, UK) and αCD8-PECy5 (Beckman-Coulter, High Wycombe, UK). This was followed by a fixing step (4% Paraformaldehyde) and permeabilisation with 0.5% Saponin. Intracellular Interferon (IFN)-γ was detected with αIFN-γ-FITC (BD Biosciences). Analysis was done on a Becton-Dickinson LSRII flow cytometer and FlowJo software.

### HLA-peptide tetramer complexes and staining for flow cytometry

All peptides used were synthesized commercially by Alta Biosciences (Birmingham, U.K.). Peptides incorporated in the tetramers were: HLA-A1 restricted epitope YSEHPTFTSQY (pp65), HLA-A2 restricted epitopes NLVPMVATV (pp65) and VLEETSVML (IE-1), HLA-B7 restricted epitopes TPRVTGGGAM and RPHERNGFTVL (both pp65) and HLA-B8 restricted epitopes ELKRKMIYM and QIKVRVDMV (both IE-1). HLA-peptide tetramers were synthesized as described elsewhere with minor modifications [26]. Tetramerisation was carried out using Streptavidin-APC (Invitrogen, Paisley, UK). To identify virus-specific CD8+ T cells by flow cytometry 1x10^6^ PBMCs were stained with tetramer at 37°C for 15 minutes, followed by staining of surface markers. Cells were then analysed on a Becton-Dickinson LSRII flow cytometer and using DIVA-software.

### HLA tissue typing of subjects

To identify which HLA-peptide tetramer complexes to use for each donor, DNA typing for the appropriate HLA-alleles was performed by PCR technique as described previously [27]. Each donor was tested for expression of HLA-A1, HLA-A2, HLA-B7 and HLA-B8.

### Statistical analysis

Data was analysed using GraphPad Prism5. The Mann-Whitney U test was used to derive p-values when comparing data between the different donor groups.

## Results and Discussion

Initial studies determined the influence of acyclovir treatment on the magnitude of the CMV-specific T cell response against two immunodominant proteins. In particular, peptide pools of the immediate early 1 (IE-1) and pp65 protein were generated and used to stimulate PBMC. These proteins were chosen as they are two of the most immunodominant antigens within the CMV proteome and represent different stages of the virus life cycle. IE-1 is expressed very early during viral replication whilst pp65 is classified as a late protein. The frequency of T cells producing Interferon-γ in response to peptide stimulation was assessed.

**Fig 1:**
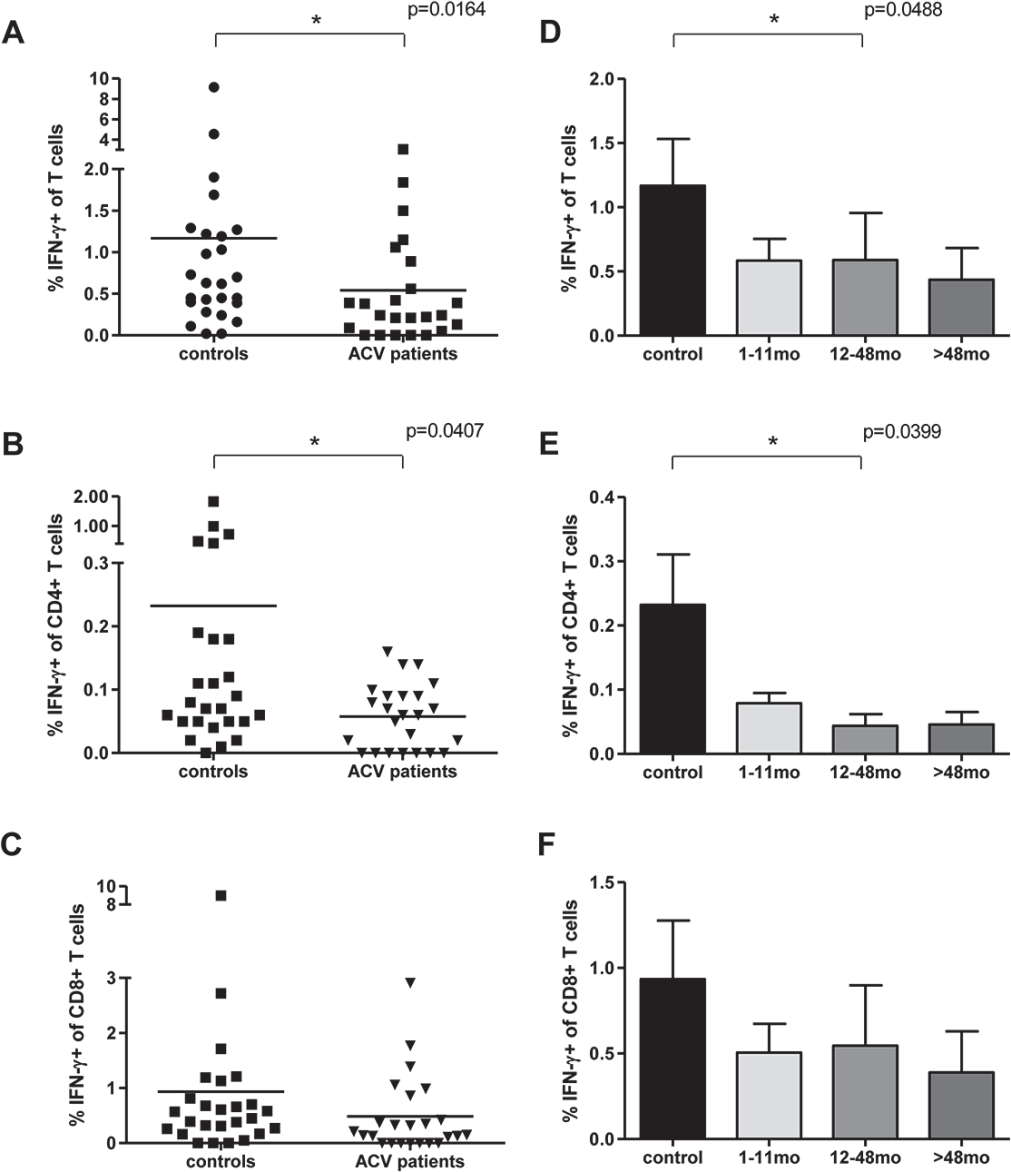
Frequency of pp65-specific T cells in patients taking acyclovir and in control subjects. IFN-γ production was determined in the total T cell population following stimulation with pp65 peptide pool using PBMC taken from ACV-treated subjects and control subjects (A). The individual responses of CD4+ T cells (B) and CD8+ T cells (C) are also shown. In (D-F) the proportion of pp65-specific T cells is represented accordingly in samples from donors who had been taking acyclovir for periods of 1–11 months, 12–48 months or over 4 years (n = 9, 8, 7 respectively). Means (A-C) or means with SEM (D-F) are shown.

The magnitude of the overall T cell response to pp65 was 1.17% in the control group and this was reduced by 53% to a mean of 0.54% of the total T cell pool in donors receiving acyclovir (Fig 1A). This reduction was seen within the first year of treatment (Fig 1D). When looking in more detail it was observed, that the CD4+ T cell subset (Fig 1B) was more affected by the treatment than the CD8+ T cells (Fig 1C). In the control group on average 0.23% of CD4+ T cells were producing IFN-γ in response to pp65-derived peptides, whereas only 0.06% responded in the treated group (Fig 1B). Relating these results to the time on treatment we did not observe significant differences, although the IFN-γ response of CD4+ T cells was more reduced in patients who had taken the drug for at least 12 months (Fig 1E).

The magnitude of the CD8+ T cell response against pp65 was also decreased in those on anti-viral therapy, but to a lesser extent. The mean CD8+ T cell response was 0.93% in the control group and this fell to 0.48% in patients on acyclovir. This trend was observed at all three time points of treatment duration, but did not reach significance (Fig 1C and 1F).

**Fig 2:**
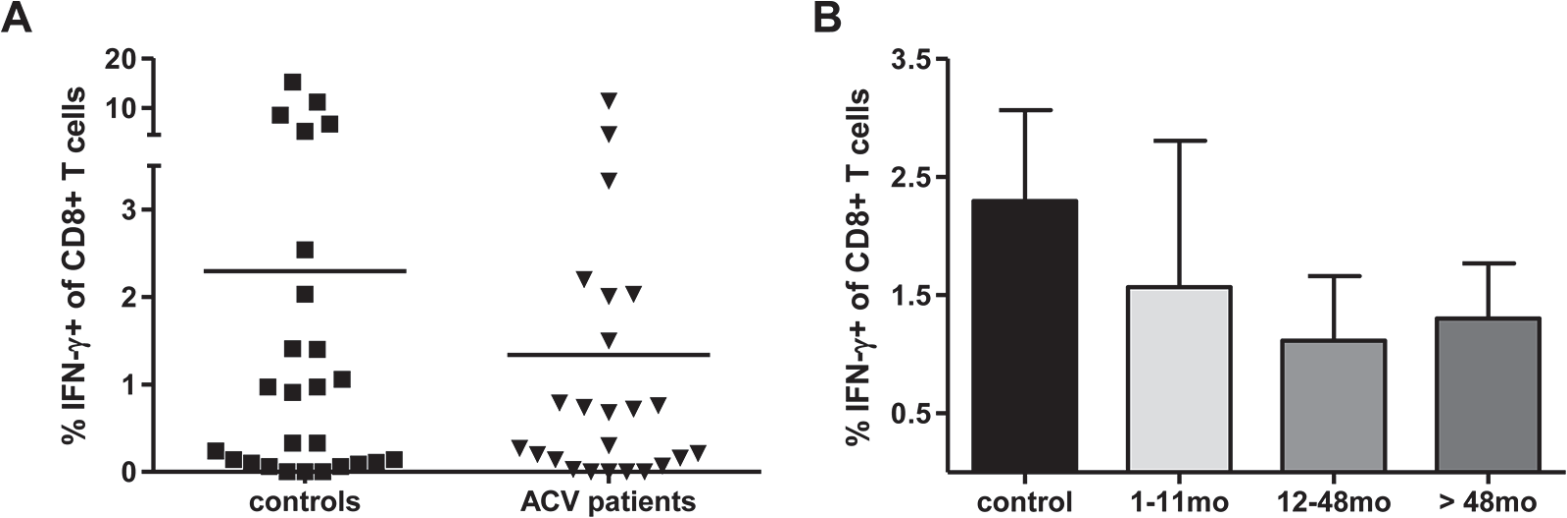
Frequency of IE-1-specific T cells in patients taking acyclovir and in control subjects. (A) Interferon-γ production was measured in response to stimulation of PBMC with IE-1 peptides in controls (n = 26) and ACV-treated donors (n = 24). (B) The frequency of IFN-γ producing cells is shown in relation to the duration of anti-viral treatment. The mean (A) or mean with SEM values (B) are shown on the figure.

We next went on to determine the T cell immune response to the IE-1 peptide pool in both cohorts. The IE-1-specific T cell response represented 2.2% of CD8+ T cell pool in the control group and was reduced to an average of 1.3% in those on anti-viral therapy (not significant) (Fig 2A). The IE-1-specific response was dominated by CD8+ T cells and the influence of acyclovir was independent of the duration of the treatment (Fig 2B). We note that for both the CD4 response to pp65 and the CD8 response against IE-1 a cluster of high responders is observed in the control group, not the ACV patients. However, these are not observed in the same individuals and we did not identify any additional features that might separate these two subpopulations. Therefore we interpret the data as a reflection of biological variation. We also stimulated PBMC with CMV-lysate, which is a mixture of proteins produced throughout the viral life cycle, and no differences were observed between the control group and the patient population using this approach (data not shown).

The differential effect of acyclovir on the immune response to pp65 or IE-1 may reflect the fact that acyclovir acts relatively late within the viral replication cycle and expression of late proteins such as pp65 would therefore be suppressed to a greater extent. In contrast IE-1 is expressed before the viral polymerase and its production is less likely to be influenced by acyclovir. The lysate contains antigens from several stages of the virus life cycle and it is possible that the reduction in the response to pp65, and potentially other proteins from the late phase of replication could be masked.

It should be noted that the doses used in our patients are below those used to suppress CMV reactivation in patients undergoing renal transplantation and therefore might be sub-optimal for suppression of CMV replication [28, 29]. However lower doses of acyclovir may be sufficient to suppress CMV replication in immune competent donors and we do not know of studies which have yet addressed this issue. Interestingly, murine CMV studies indicate sporadic events leading to transcription of IE transcripts during latency which may lead to reactivation [30]. Low dose ACV treatment may enhance termination of replication at this early checkpoint and therefore enhance the stop of such stochastic reactivation events. It has also recently been reported that the use of valacyclovir at the dose of only 500mg daily can reduce the number of Epstein-Barr virus (EBV)-infected B cells in healthy donors [31]. Unfortunately we were, as anticipated, not able to detect CMV DNA in our healthy donors and so cannot directly monitor the effect of treatment on CMV viral load [32].

**Fig 3:**
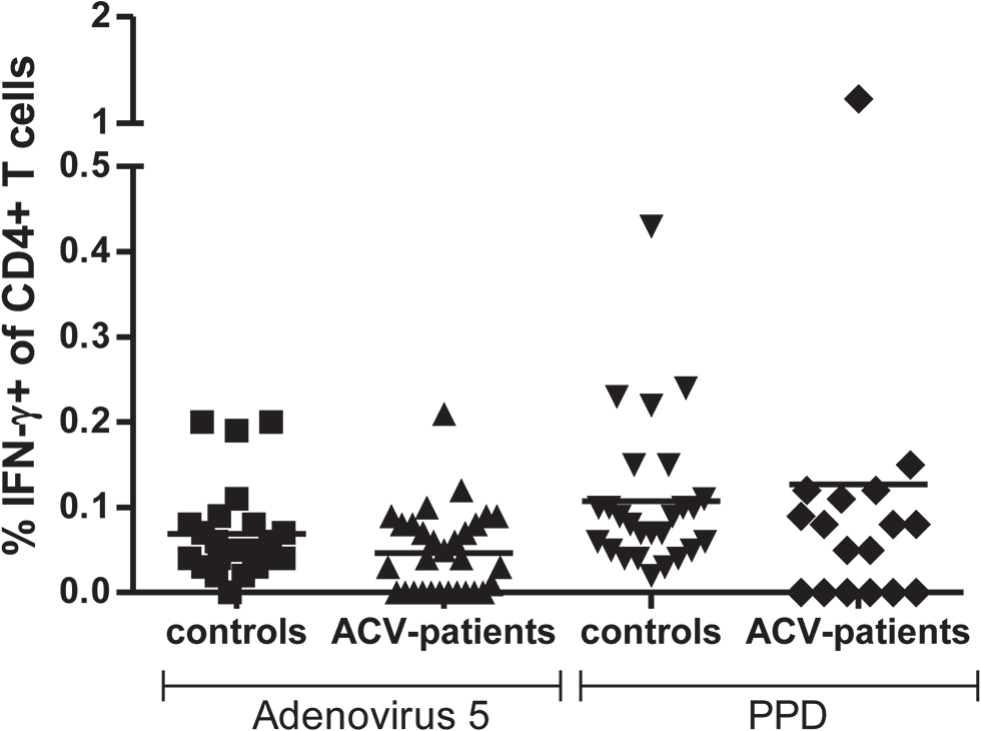
CD4+ T cell response to control antigen in patients taking acyclovir and in control subjects. PBMC from control subjects (n = 26) and donors taking acyclovir (n = 24) were stimulated with Adenovirus 5 or PPD antigen and the proportion of IFN-γ producing T cells was determined using intracellular cytokine analysis. Horizontal bars represent means.

In order to demonstrate that acyclovir has no effect on general immune function we went on to measure the immune response to two control antigens in both cohorts. Adenovirus 5 was used as a source of viral protein and tuberculin purified protein derivative (PPD) was used as an additional antigen. The T cell IFN-γ response against both of these was not altered by ACV treatment (Fig 3).

**Fig 4:**
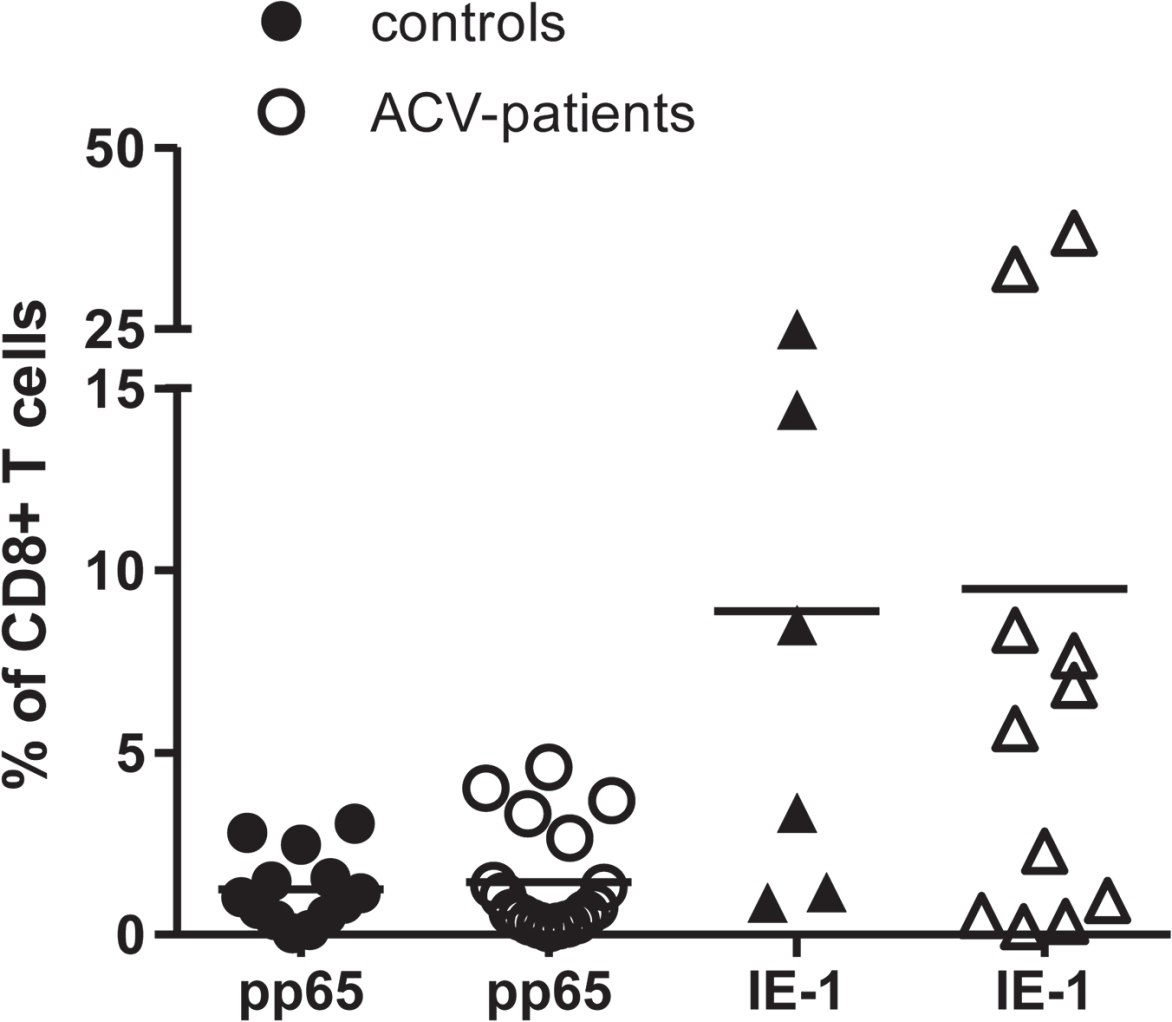
Frequency of pp65 and IE-1-specific CD8+ T cells detected by HLA-peptide tetramer staining in control subjects and donors taking acyclovir. PBMC were stained with HLA-peptide tetramers specific for pp65- and IE-1-derived epitopes according to the individual HLA genotype. The aggregate frequency of pp65 or IE-1-binding T cells was determined for each donor and is shown in the figure. Horizontal bars depict the mean frequencies.

Having demonstrated that acyclovir treatment was able to reduce the functional immune response against the late CMV protein, we then determined the absolute frequency of CD8+ T cells specific for IE-1 and pp65 using a panel of HLA-peptide tetramers. The HLA genotype of each donor was determined and the appropriate tetramers were then used to stain T cells from peripheral blood. The number of HLA-peptide binding T cells was then aggregated within each donor and is depicted in Fig 4. pp65 tetramer-binding T cells represented an average of 1.3% of the CD8+ T cell pool in untreated controls compared to 1.5% of CD8+ T cells in ACV-patients. The proportion of IE-1 tetramer-binding cells was considerably higher with values of 8.9% and 9.5% of all CD8+ T cells respectively. These data indicate that, despite having observed a reduction in IFN-γ production in response to pp65, the absolute number of pp65 and IE-1-specific CD8+ T cells was not changed by anti-viral treatment. There are a number of possible explanations for this observation including the fact that the tetramer assays only assess a very small range of those epitopes contained within the peptide pools. In addition the tetramers identified only CD8+ T cells and acyclovir treatment had a more marked effect on the CD4+ T cell response against pp65. Finally, it is possible that anti-viral therapy reduces the functional activity of CMV-specific memory cells, due to a reduction in viral antigen, whilst the pool of recirculating peptide-specific cells is not markedly altered. The function of CD4+ T cells may potentially be more affected due to less frequent T cell stimulation as the antiviral treatment will lead to reduced uptake of viral antigens by antigen presenting cells.

The reduction of the magnitude of the CMV-specific immune response may also take some time to become apparent. Recent studies have started to address the half-life of CMV-specific T cells, revealing in both human and the murine system that such cells turn over slowly with t_1/2_ in the range of 45–60 days [33, 34]. CD45RA+ memory T cells, which are a particular feature of the CMV-specific immune response, exhibit particularly low rates of cell turnover and may therefore be least affected by anti-viral medication [35]. This indicates that the time of treatment, the dose and also patient compliance will be important issues in therapy to suppress CMV-specific immunity. It is noteworthy that HIV-specific CD8+ T cells decline with an average half-life of 38 weeks after patients commenced HAART therapy and so any anti-viral treatment that is used to reduce the CMV-mediated “memory inflation” will have to be administered for considerable periods of time [20]. This is in concordance with the murine studies where a 4–6 month treatment with antiviral drugs was not sufficient to reverse memory inflation of MCMV specific immunity [21, 23]. It is also important to remember that the dose used in this study was low and the serum half-life of ACV is only around three hours [36]. The effect of anti-viral treatment in the murine system only became fully apparent after long term administration of the drug [23]. Therefore, an analysis of the ability of higher and more regular doses of acyclovir would be of interest in the future. It is also likely that the efficacy could be increased by administration of valacyclovir, the prodrug of acyclovir which has a bioavailability of up to 5 fold higher and may therefore be advantageous in future studies [37]. Toxicity of both these viral agents is low which is an important consideration in relation to their potential use for long term therapy [38]. An increased risk of morbidity and mortality has been correlated with increasing anti-CMV antibody titre and CMV-specific T cell expansions in several studies, in particular in relation to cardiovascular disease and inflammatory conditions [15, 17]. As such it might be recommended to select those individuals with large CMV-specific immune responses for long term ACV treatment. They are most likely to benefit from intervention and unnecessary drug administration for individuals which would see little benefit from such long term treatment would be avoided.

In summary we show that acyclovir therapy has the potential to reduce some components of the CMV-specific T cell response. This may prove to be of therapeutic benefit not only to older adults, but also for patients with inflammatory diseases in whom CMV may serve to increase disease severity [39, 40]. Consideration should be given towards optimisation of therapies to suppress CMV-specific immune response with the aim of improving overall immune competence.
